# Deep Learning Segmentation of Chromogenic Dye RNAscope From Breast Cancer Tissue

**DOI:** 10.1007/s10278-024-01301-9

**Published:** 2024-10-23

**Authors:** Andrew Davidson, Arthur Morley-Bunker, George Wiggins, Logan Walker, Gavin Harris, Ramakrishnan Mukundan, kConFab Investigators

**Affiliations:** 1 Department of Computer Science and Software Engineering, University of Canterbury, Christchurch, New Zealand; 2 Department of Pathology and Biomedical Science, University of Otago, Christchurch, New Zealand; 3 Canterbury Health Laboratories, Christchurch, New Zealand; 4 Sir Peter MacCallum Department of Oncology, The University of Melbourne, Melbourne, Victoria Australia; 5 Peter MacCallum Cancer Center, Melbourne, Victoria Australia

**Keywords:** Computational pathology, Image segmentation, Cancer, RNAscope, Deep learning, Machine learning

## Abstract

RNAscope staining of breast cancer tissue allows pathologists to deduce genetic characteristics of the cancer by inspection at the microscopic level, which can lead to better diagnosis and treatment. Chromogenic RNAscope staining is easy to fit into existing pathology workflows, but manually analyzing the resulting tissue samples is time consuming. There is also a lack of peer-reviewed, performant solutions for automated analysis of chromogenic RNAscope staining. This paper covers the development and optimization of a novel deep learning method focused on accurate segmentation of RNAscope dots (which signify gene expression) from breast cancer tissue. The deep learning network is convolutional and uses ConvNeXt as its backbone. The upscaling portions of the network use custom, heavily regularized blocks to prevent overfitting and early convergence on suboptimal solutions. The resulting network is modest in size for a segmentation network and able to function well with little training data. This deep learning network was also able to outperform manual expert annotation at finding the positions of RNAscope dots, having a final $\varvec{F}_\textbf{1}$-score of 0.745. In comparison, the expert inter-rater $\varvec{F}_\textbf{1}$-score was 0.596.

## Background

### Overview

According to the World Health Organization (WHO) data from 2020 [1], breast cancer was the world’s most widespread form of cancer with 7.8 million women diagnosed over the past 5 years. If diagnosed early, treatment can be highly effective for patients. Various cancer features including oestrogen receptor status, progesterone receptor status, HER2 status, and intrinsic subtype (e.g. basal, luminal A, luminal B, normal-like) give insight into which treatments will be effective [2]. Therefore, it is important for these features to be classified reliably to improve patient outcomes. The number of cancer cases in recent years has been increasing and is projected to continue doing so [3], placing further pressure on pathology services, which are already under significant strain [4]. Fewer pathologists coupled with a growing workload could negatively impact the efficiency of patient care. There is a growing need for new tools that can help pathologists rapidly diagnose patients within the healthcare system.

Presently, pathologists will generally examine small areas of interest from each tissue slide as examining all the available tissue is labour intensive and time consuming. With the introduction of whole slide imaging [5, 6], there is a wealth of high quality tissue slide data available. When combined with proper ground truth annotations, this provides the data needed to train deep learning quantification methods that could ease the pathology workload. It should be noted that the methods presented in this paper are experimental and are for Research Use Only (RUO).

**Fig. 1:**
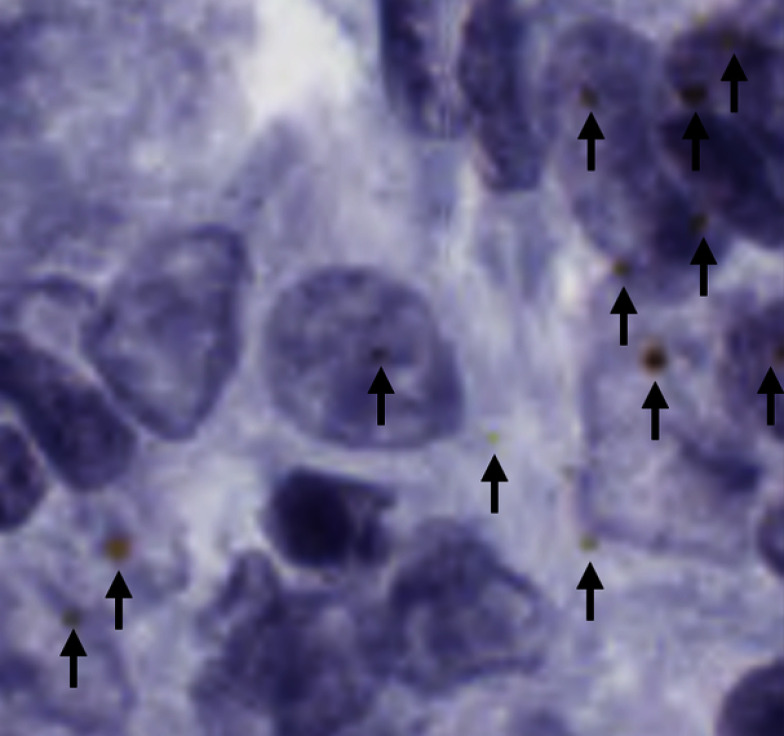
A small area of haematoxylin and chromogenic RNAscope stained breast cancer tissue, showing RNAscope dots (annotated with black arrows) with varying shape, size, and hue

### RNAscope

RNAscope is an RNA *in situ* hybridization assay that can be used to visually assess the level of gene expression in a tissue sample [7]. The RNAscope probes bind to RNA containing a user-defined gene sequence, and then an amplifying detection reagent produces a visible stained dot at the site of hybridization. These dots represent single RNA transcripts that have been detected by RNAscope. Practically, this means that RNAscope staining will produce tissue samples containing coloured dots, and the number of dots (or transcripts) per nucleus indicates the level of expression of whichever gene was chosen. This makes it useful for breast cancer diagnosis, since some of the aforementioned statuses can be found by assessing the expression of specific genes in the cancer tissue. For example, the level of expression of the Erb-B2 Receptor Tyrosine Kinase 2 (*ERBB2*) gene, also referred to as *HER2*, is an important indicator.

This study focuses on the chromogenic RNAscope dye (3,3‘-Diaminobenzidine, DAB), as this detection method allows for standard bright-field microscopy evaluation that is commonly used by pathologists. Chromogenic RNAscope dye can simply be used with haematoxylin staining which helps to identify morphological structures within a tissue section, including cell nuclei. The stained tissue section can then be scanned under normal laboratory conditions to produce a slide image.

The most difficult and time-consuming cases to assess and quantify gene expression on are those that contain little to no RNAscope staining. Intense staining is easy to detect and clearly shows high expression of the target gene(s). Therefore, this study focused on areas of tissue with little to no RNAscope staining. This made the segmentation task more difficult for a few reasons; these images had very low representation of the positive class (RNAscope stained areas), the RNAscope dots varied in size, colour, and shape (seen in Fig. 1), and the amount of annotated data was small. Therefore, the target segmentation method had to be able to function with little training data, be sensitive to an underrepresented positive class, and be sensitive to structures of varying characteristics.

This paper covers the development of a deep learning segmentation method to accurately quantify RNAscope staining in breast cancer tissue. Our motivation was to produce a robust method that was not calibrated around the best quality tissue, and that was not limited to RNAscope signals that were well defined and intense. As such, this study used archival FFPE (formalin fixed paraffin embedded) tissue samples that researchers might typically encounter, which may contain compromised RNA. This meant that the stained images incorporated a wider range of signal intensities. The tissue samples were stained with chromogenic RNAscope and haematoxylin.

## Related Work

ConvNeXt [8] is a family of convolutional neural network architectures which are built for classification. They are an improvement on the ResNet architecture, using design cues from vision transformers, which are another type of network architecture that were state of the art at the time. ConvNeXt is fully convolutional, making it comparatively simple and efficient compared to vision transformers.

U-net++ [9] is a deep learning segmentation network architecture. It shares the same general ‘U’ shape as a U-net with downsampling layers on the left side of the ‘U’ and upsampling layers on the right. However, U-net++ modifies the bridging skip connections between the downsampling and upsampling layers by adding additional upsampling pathways that are heavily interconnected. These additional pathways aim to decrease the semantic gap between the downscaling backbone and upscaling layers and, in doing so, make it easier for useful features to make it into the final output layers without being lost.

Morley-Bunker et al. recently conducted a study [10] comparing existing methods for quantifying chromogenic RNAscope staining from digital slide images of colorectal cancer tissue. These tissues were also stained with haematoxylin, to give definition to the nuclei. Most of the methods compared in the study were not fully automated and required the user to either verify every RNAscope candidate or manually select RNAscope positions. The open-source method that required the least user input was Trainable WEKA Segmentation [11], but this was still not considered fully automated in the study due to the number of user steps required. It also could not reliably isolate individual RNAscope dots, sometimes outputting clusters instead. The closed-source, commercial methods tested also required user configuration to function and did not outperform the open-source methods by most metrics assessed.

**Fig. 2:**
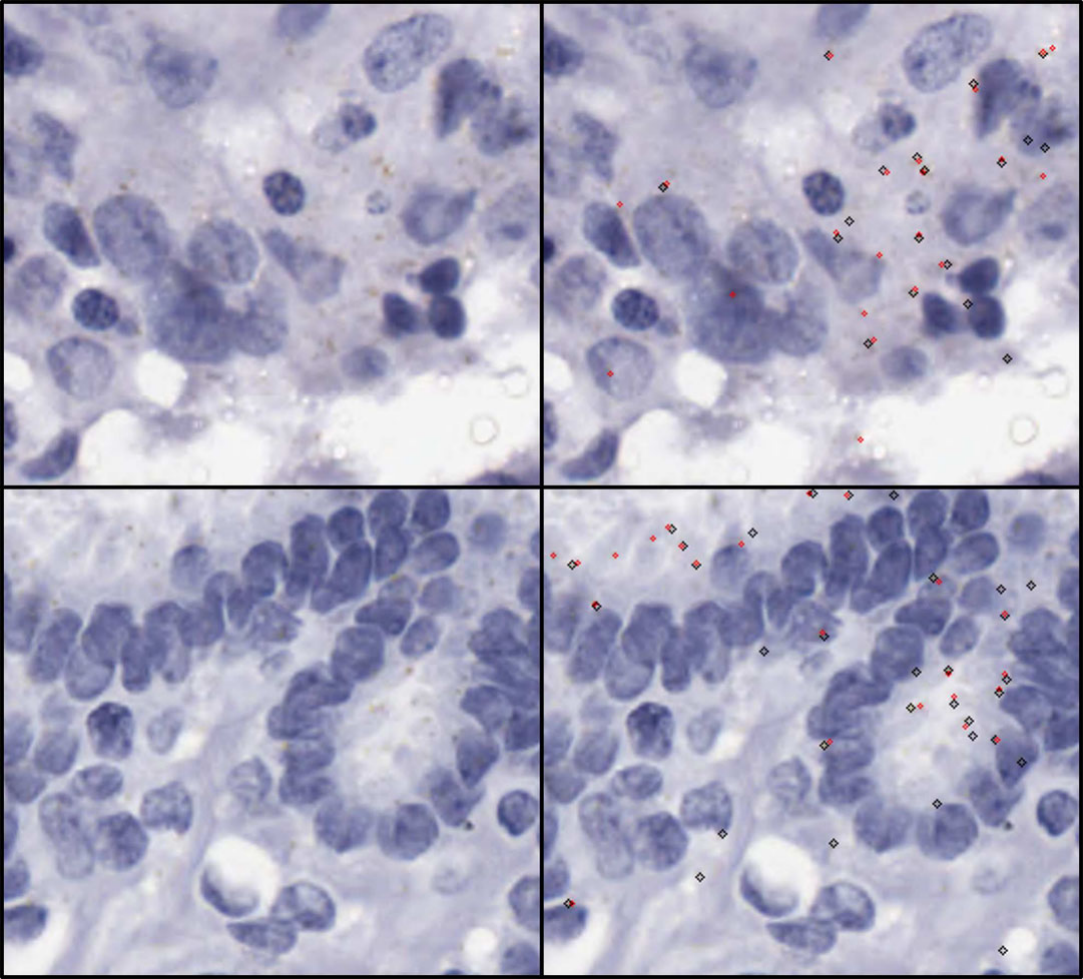
This figure contains a visualization of expert annotations that were used to assess manual annotation agreement. The black diamonds represent one set of annotations, and the red diamonds represent the other. Each tissue region is shown with no annotations on the left side and with annotations on the right side

Jamalzadeh et al. developed QuantISH [12], which is a method for quantification of chromogenic RNAscope and haematoxylin stained tissue. It works by firstly using colour deconvolution to separate the brown chromogenic RNAscope stain from the blue haematoxylin, which relies on set colour values for each stain. The brown RNAscope channel is masked, and the resulting regions are expanded. This RNAscope mask is then used to measure the RNAscope intensity in each cell. This method allows for quantification of gene expression at a cell-by-cell basis, but does not isolate the RNAscope staining into individual dots, which are a more exact indicator of the level of gene expression.

Davidson et al. recently published a grey level texture feature-based method for chromogenic RNAscope segmentation [13]. This method was able to perform similarly to manual expert annotation. Given the high level of difficulty of this problem, we thought it was worth developing a more nuanced deep learning method. Aside from the grey level texture feature method, the existing solutions for chromogenic RNAscope segmentation are either not sufficiently automated to be convenient, do not allow for individual dot counting, or are closed-source with no published, peer-reviewed accuracy metrics.

## Method

### Data Acquisition

A total of 40 stained whole slide images of tissue microarrays were obtained, with each consisting of 26 to 116 tissue cores. The tissues were scanned at 40x magnification (0.25 microns per pixel). All of these samples used the brown chromogenic RNAscope stain colour and were provided in the Aperio (.svs) format. Each tissue core on each tissue microarray was extracted from a different tissue block, each of which were from different patients. This meant that every tissue core was from a different patient, with the tissue sample having varying age and storage conditions, which contributed to the variability of the dataset. A control probe for the bacterial gene dapB was used to confirm absence of staining with negative control TMA sections.

**Fig. 3:**
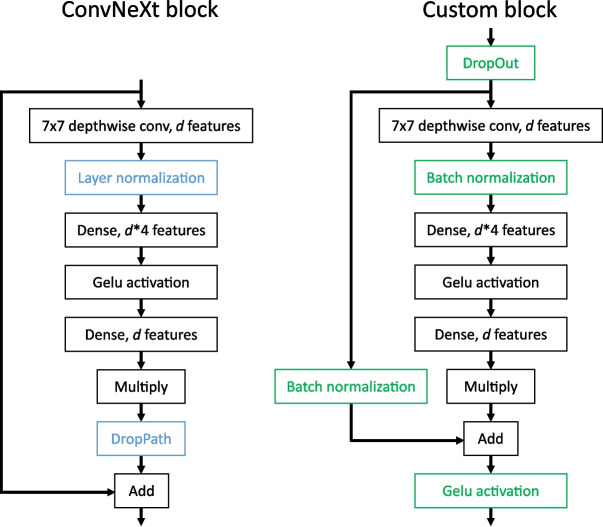
A ConvNeXt block (left) compared to the custom upscaling block used in this paper (right). The differing layers are coloured. Batch normalization and activation layers were added in the custom block, which also uses DropOut regularization instead of DropPath

### Expert Annotation of RNAscope Dots

The same expert annotations from the previous study by Davidson et al. on using grey level texture features for RNAscope segmentation [13] were used in this study. The dataset provides both a baseline inter-rater agreement metric to compare against and a limited amount of annotated training data. Specifically, there are a total of 144 480x480 pixel non-overlapping patches of haematoxylin and chromogenic RNAscope stained tissue annotated with RNAscope positions. 133/144 of these patches are from different patients. The annotations were done by a trained pathology scientist and an anatomical pathologist, with an overlap of 19 patches annotated by both, which contained a total of 472–607 dots by each annotator. A sample of the two sets of overlapping annotations is shown in Fig. 2. The agreement between the two experts with 5 pixel tolerance was assessed to be 0.596. This score may appear low, but it is expected due to the difficult nature of the problem and the fact that annotations must both be matching and be very near each other to count as true positives. Due to the coordinate format of the ground truth data, post-processing will be done on the segmentation maps produced by the deep learning networks investigated later in this paper to convert them into a comparable format.

### Overview

We developed a deep learning segmentation network architecture to accurately segment RNAscope dots. Multiple design considerations were made to account for the small dataset, including the use of regularization layers, feature throughput in the network, and training data augmentation. The backbone (downsampling section) of the network is an implementation of ConvNeXt [8], while the upscaling layers instead use a custom convolutional block that has been designed for strong regularization to facilitate convergence even with small datasets that suffer from heavy class imbalance. The overall network structure partially follows the U-net++ [9] pattern, with some adjustments to fit the network design of the ConvNeXt backbone. We also developed an artificial data generation pipeline. All functionality was implemented in Python, mainly using the Tensorflow [14] and Keras [15] libraries.

### Backbone

The backbone of the network is an implementation of ConvNeXt. The amount of convolutional blocks at each resolution follows the ConvNeXt-base specification, which gives an intermediate level of depth. The proposed network architecture in this paper would work for any size specification of ConvNeXt; the ‘base’ size was simply chosen as it was the largest backbone size that would allow the entire model to function with batch size 4 within 8GB of VRAM, as this matched the testing environment.

**Fig. 4:**
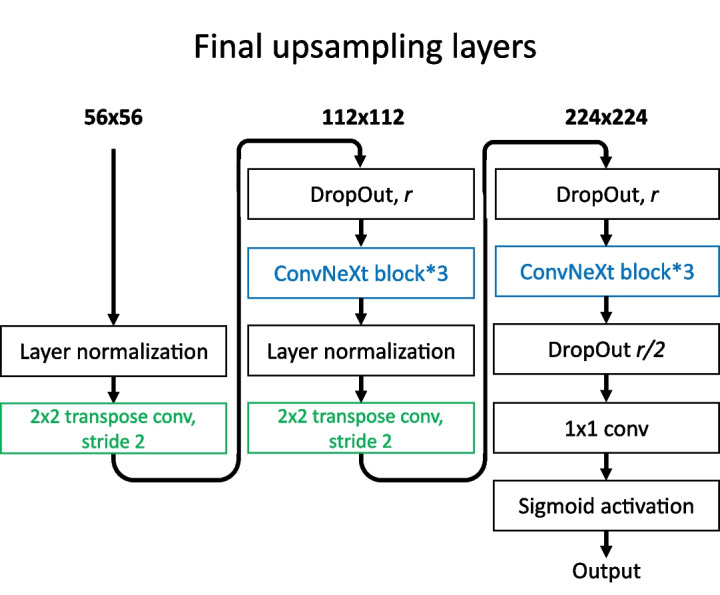
The final upsampling section of the network architecture detailed in this paper, which includes the network output. The upsampling layers are shown in green, and the ConvNeXt blocks are shown in blue

### Upscaling Layers

The upscaling layers of the network use a custom block which is based on the ConvNeXt block, but modified to increase regularization while still allowing for convergence. Each upscaling layer is preceded by only a single convolutional block, as their purpose is largely to organize incoming features; most of the advanced feature extraction should be done in the backbone, which contains more convolutional blocks. Upscaling regularization was done to provide robustness against any single feature in the network overfitting to the limited training data, as these features could freely move through the skip connections and upscaling layers to the end of the network otherwise. Since there is only one block before each upscaling layer compared to the 3–27 blocks before each downsampling layer, DropPath (stochastic depth regularization which randomly skips entire blocks) as proposed by the original ConvNeXt paper [8] is potentially less effective for the upscaling blocks. The output changes caused by the entire convolutional block before an upscaling layer being skipped on some iterations would likely destabilize the learning surface. For this reason, DropOut, which operates on a feature-by-feature basis, was also investigated as an alternative to DropPath for these upscaling blocks. When enabled, it is applied before the skip connection branch (as seen in Fig. 3) as opposed to DropPath, which is applied only to the convolutional layers in the ConvNeXt block. The usage of DropPath in a wider scope necessitated some further changes to the block to allow for convergence. The layer normalization layer was switched to batch normalization, and a second batch normalization layer was added along the skip connection path. Batch normalization is applied across the batch axis, whereas layer normalization is applied along the feature axis as described in [16]. This change was adopted since the network would not converge using layer normalization, but it would converge with batch normalization. Although batch normalization has the drawback of being dependent on batch size, which must be small in segmentation networks due to memory constraints, we speculate that it performs better in this case since it directly represents the differing distributions (mean and standard deviation) for each separate feature, whereas layer normalization first normalizes across the feature axis, which has the effect of squishing the outgoing values for features that have lower standard deviation than the other features. Even though layer normalization does introduce trainable bias and gain which would theoretically offset this disadvantage with the correct weights, it requires training of a second set of weights to do so and therefore makes it more difficult for convergence to occur. However, to be certain, more detailed analysis would be required. Finally, an additional activation function was added at the very end of the block to account for the additional batch normalization layer added to the skip pathway. The resulting block can be seen in Fig. 3, alongside the original ConvNeXt block it was based on. Either DropPath or DropOut can be enabled on this modified block.

**Fig. 5:**
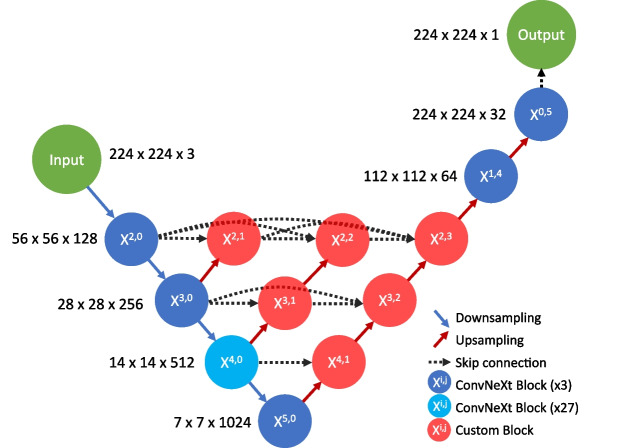
The custom U-net++ based architecture that is described in this paper. It uses a ConvNeXt backbone, which is attached to heavily interlinked, custom blocks (red) that filter and upsample the outputs from multiple points in the backbone. The final layers use ConvNeXt blocks between the last two upsamples, ending with an output segmentation map

**Fig. 6:**
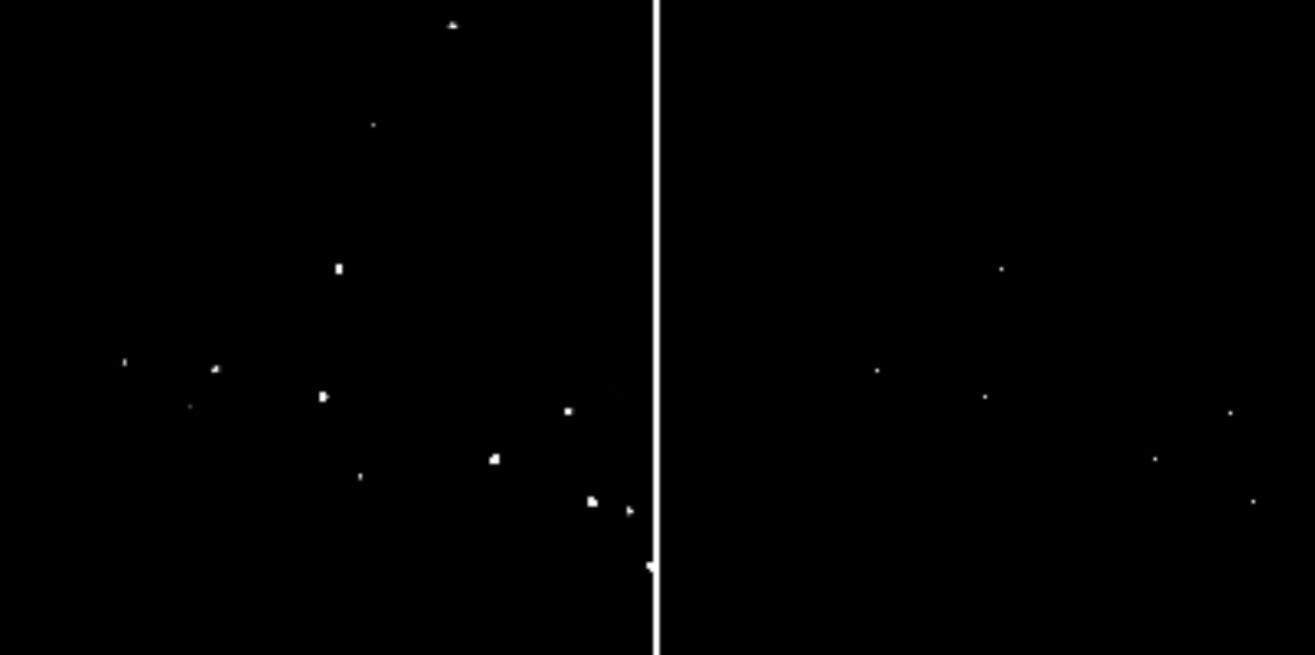
An example of the input (left) and output (right) of the post-processing applied to the raw outputs from the deep learning network. Binarization with a configurable grey threshold is applied, and then the watershed transform is used to reduce the remaining pixel clusters into singular dots, provided that their area meets the area threshold

### Overall Structure

The overall network is partially structured based on the U-net++ architecture [9], which is a more heavily interconnected variant of U-net. To apply this architecture effectively with a ConvNeXt backbone, some changes were made. ConvNeXt begins with a 4x4, 4 stride convolution, which results in downsampling by a factor of 4. The U-net++ architecture is only applied past this point, as it significantly reduces the amount of parameters to train, allowing for more depth in other parts of the network. It also circumvents the issue of requiring several expensive and difficult to train 4x upsampling layers. We hypothesized that only applying the U-net++ architecture to the deeper layers would still retain much of the ability to facilitate better feature movement through the network, as the most important and large-scale features reside in the deeper layers of the network. This design means that deep supervision as proposed in the U-net++ paper is not viable, as there is only one full-resolution output. To reach a full resolution output from the end of the nested layers, the output must be upscaled by a factor of 4. Since learning to upscale 4x to an accurate segmentation with no incoming skip connections is a difficult task with a large semantic gap, more convolutional blocks were added to this part of the network compared to the rest of the upscaling layers. The structure is similar to the downsampling portion of the network; two times over, the output is passed through 2D transpose convolution with a stride of 2 to double the resolution, followed by 3 ConvNeXt blocks. For this section, DropPath can be optionally applied within the ConvNeXt blocks, or DropOut can be applied in between them. The exact structure of the final 4x upsampling layers can be seen in Fig. 4. The full network structure can be seen in Fig. 5.

The resulting network aims to incorporate the efficiency and good feature representation of ConvNeXt with the improved feature propagation of U-net++ into a single, heavily regularized network. This network is therefore applicable to medical segmentation tasks that require heavy regularization due to class imbalance and/or a low amount of quality, annotated training data.

### Post-Processing

The raw output from the deep learning network most often contained detections that were made up of clusters of multiple pixels. The desired output format was a single co-ordinate value for each RNAscope dot, which could then be directly compared against the ground truth. Therefore, post-processing operations were added to convert the segmentation maps outputted by the deep learning segmentation network into individual RNAscope dot co-ordinates. An example of the input and output of this process is shown in Fig. 6. Firstly, a binary threshold with a configurable threshold value (referred to later as the grey threshold) is applied to the segmentation map to reduce the number of unique values in the image to two, so that the watershed transform can be subsequently used. Then, the watershed transform is applied to find each cluster of pixels and extract their central co-ordinates to use as RNAscope dot positions. The connected-pixel threshold (thereafter referred to as area threshold) of the watershed transform, which controls how many pixels must be in a cluster for it to be detected, was left configurable. Further discussion of the grey threshold and area threshold values is included in Section “Results and Discussion” (Results and Discussion).

**Fig. 7:**
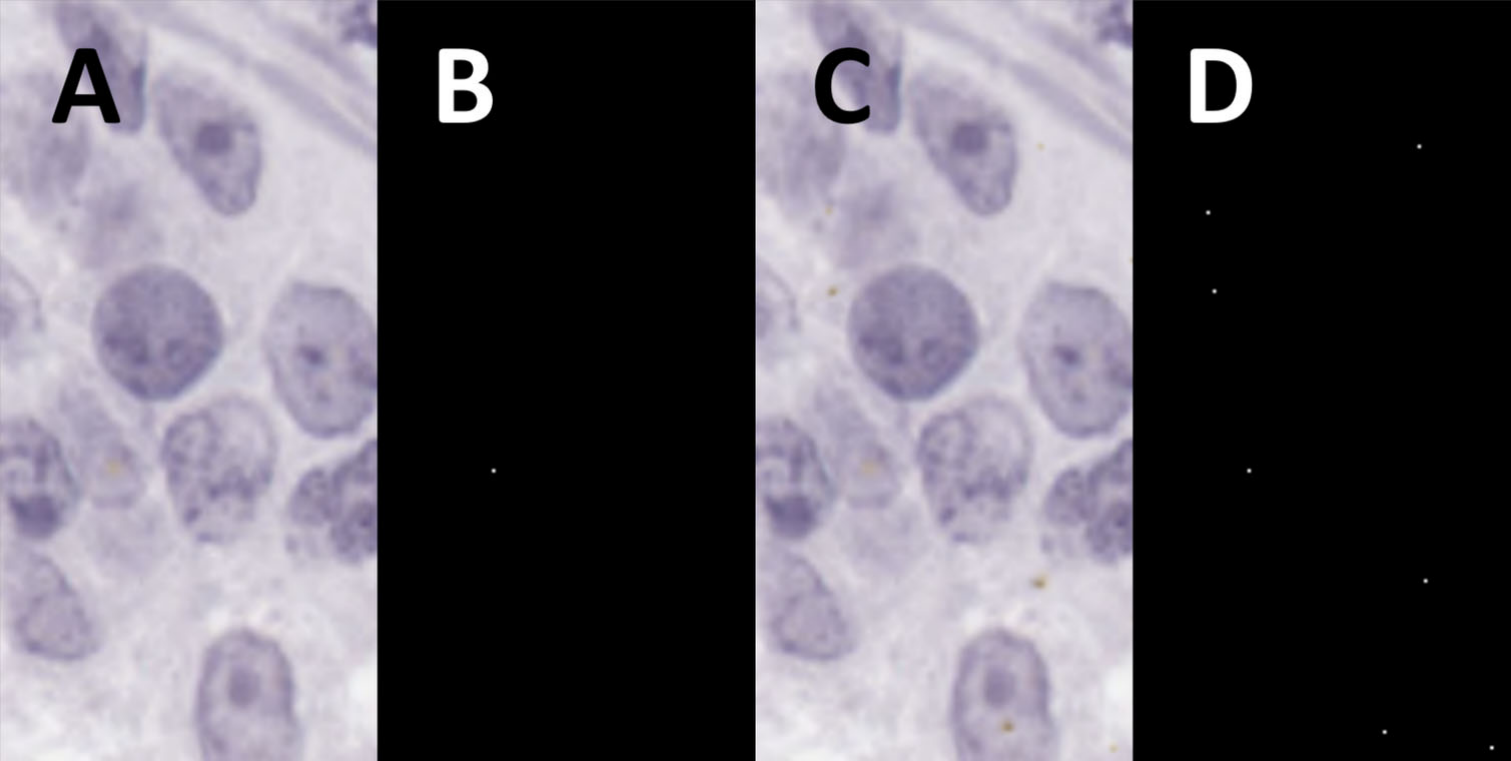
The main steps in the process for generating additional RNAscope segmentation training data. **A** shows an input image which has not undergone any processing yet. **B** shows the RNAscope dots from the input image that were detected and annotated using a texture feature-based method [13]. **C** shows the image once multiple, artificial RNAscope dots have been added using image processing techniques that are described in this section. **D** shows how the positions of the generated dots are recorded with perfect accuracy, providing many additional RNAscope dots for use as training data

**Fig. 8:**
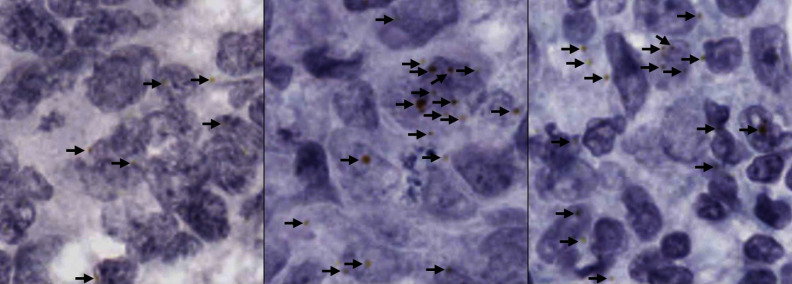
An assortment of small patches showing the varying appearance of RNAscope dots, which are annotated with black arrows. In the leftmost image, the RNAscope dots are pale, yellow circles. In the middle image, there is both pale yellow circles (bottom left) and darker dots in the centre. The rightmost image shows RNAscope dots that present as a dark dot with a yellow tinge around their edges

### Data Generation

A texture feature-based RNAscope segmentation method developed by Davidson et al. [13] was used in combination with image processing techniques to generate additional training data for use in network training, since there were only 144 patches properly annotated with ground truth data. The texture feature-based segmentation method was used to annotate the existing, natural dots on the patches. It works by extracting a set of grey-level texture features around each potential candidate pixel and then evaluating the resulting feature vector using a support vector classifier which was trained to detect RNAscope dots. This gives a prediction for each pixel, which can then be collated into a segmentation map. Processing of the resulting segmentation map to obtain RNAscope dot co-ordinates is discussed in the following paragraph. Image processing techniques were used to add additional RNAscope dots, which, by virtue of being artificially added, could also be annotated with full accuracy. The method was applied to a total of 1071 candidate patches that had been identified for potential expert annotation but did not end up being annotated. Figure 7 shows the major steps in the data generation process.

As the first step in the data generation process, the feature-based RNAscope segmentation method [13] was applied to the candidate patches to annotate any dots that already naturally existed in the patches. It uses grey-level texture features to predict which pixels in each patch contain RNAscope dots, producing a segmentation map. For post-processing of the raw segmentation maps, a similar approach to that described in Section “Post-Processing” (Post-Processing) was taken. A 5x5 circular Gaussian blur was first applied, and then a binary threshold at a cut-off value of 148. The watershed transform was then used to find individual RNAscope dot co-ordinates, with area threshold set to 0. This process was configured to be highly selective to mitigate the possibility of many false positives making it into the generated data. Following this, extra dots were added to each patch. This was done since the RNAscope dots are normally not visually complicated, but are often indistinct against a complicated background which leads to the segmentation problem being difficult. The aim was to replicate two common types of RNAscope dot: a simple radial dot that is yellow or brown and a dark dot with yellow tinges around the edge. Real examples of both types of these dots are shown in Fig. 8.

**Fig. 9:**
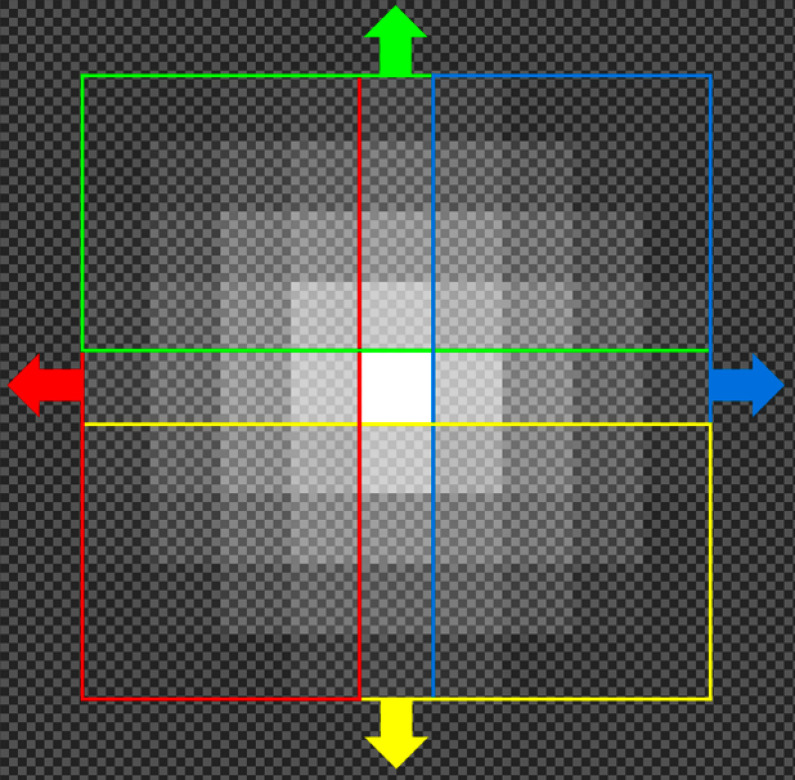
A demonstration of the areas effected by cardinal scaling during artificial dot creation. Each of the four directions, which have a starting radial length of 4 pixels, is randomly scaled to extend by 0–3 pixels

**Fig. 10:**
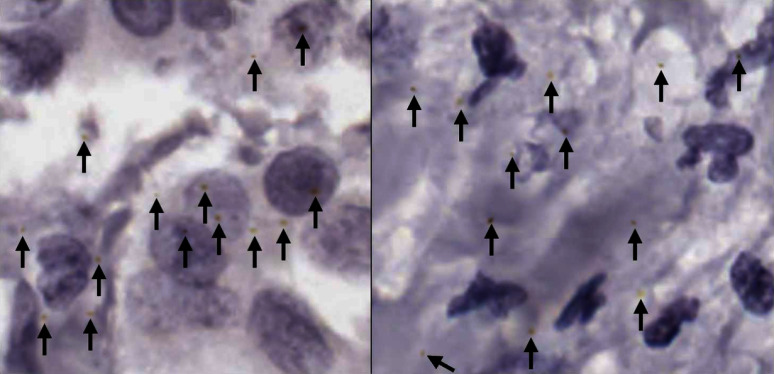
Artificially generated RNAscope dots (annotated with black arrows). The left image shows only the first type of generated dot without a dark centre, and the right image shows both types of generated dot

The dots are created by firstly initializing an empty 3-channel (BGR) image, which will be used as a subtractive mask that can be applied over a region of a patch. The mask values are initialized to 0. A random colour between light yellow (225, 253, 255) to dark brown (160, 200, 217) is calculated using linear interpolation, with the same random value being used to interpolate all 3 channels. The centre pixel of the mask is set to the opposite of the selected colour ($255 - value$ on each channel) to allow it to be applied subtractively. Next, a circular Gaussian blur with kernel size 9x9 pixels is applied, which gives the basic desired fade out pattern but considerably darkens the dot. To counteract the darkening, the values in the mask are multiplied such that the central pixel regains its initial value before the blurring. If this dot is meant to replicate the dark type of dot from Fig. 8, the same process of creating, blurring (using a smaller 5x5 pixel circular kernel), and multiplying a dot is followed with a new mask. The colour (200, 210, 215) is used instead to give significant darkening of at least 40 on each channel. This secondary mask is then added to the first mask, with a random x and/or y offset of -1 to 1 pixel to replicate the commonly off-centre nature of the yellow tinges behind this type of dot.

Finally, random perturbations are applied to the dot to represent the variation in real dots more accurately. Each cardinal direction from the centre dot is randomly stretched by 0–3 pixels as shown in Fig. 9, the entire dot is scaled by an x and y factor (described in the following sentences), some random noise which is randomly selected from 0.975–1.025 for each pixel is multiplied onto each colour channel, and lastly the dot is rotated by a random integer angle (0°- 359°). The scale factor is calculated as a random number from 0.25–0.5 separately for the x and y axis, with a single random number $r'$ calculated according to Eq. 1 added to both. The reason for adding a squared uniform random value (which will most frequently be a low value) to both dimensions is to represent the low frequency of cases where a substantially larger dot appear in real images. Some examples of the resulting dots can be seen in Fig. 10.

1 $$\begin{aligned} r'= &   0.4r^{2}~\text {where}~\textit{r}~\text {is a uniform random}\nonumber \\  &   \text {number from 0 to 1.} \end{aligned}$$

Since the characteristics of dots within a patch tend to be similar, the generated dots are designed to follow a similar pattern. Therefore, some values for parameters involved in creating these dots are controlled separately for each patch. Although the primary colour is randomly selected from yellow to dark brown (as previously defined), each patch is only allowed to select from a randomly selected, continuous 20% of this range. There is a 25% chance of the secondary (dark-centred) dot type being present in the patch; otherwise, there will be none at all. If the patch is selected to have these secondary dots, the probability of each generated dot being of the secondary type is randomly selected to be 0–50%. To represent how most patches have either few dots (0–5) or many dots (20+), the number of dots to add for a patch, *n*, is calculated according to formula 2 which gives an exponential scale from 2 to 64. The dots are placed at random x and y co-ordinates on the patch, with the random x and y values being recalculated until the co-ordinate is at least 3 pixels away from any other existing dot.

2 $$\begin{aligned} n= &   round(2^{1 + 5r})~\text {where}~\textit{r}~\text {is a uniform}\nonumber \\  &   \text {random number from 0 to 1} \end{aligned}$$

**Fig. 11:**
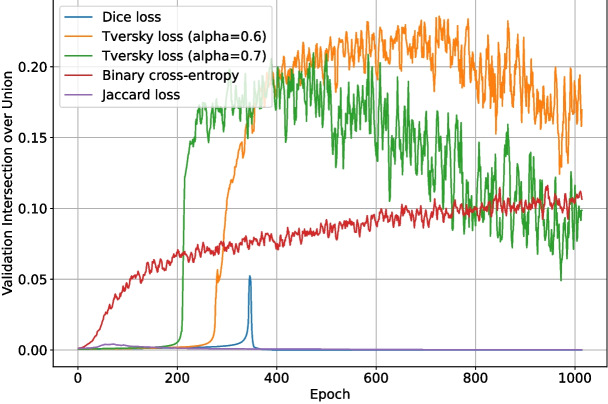
The validation intersection over union during training of the deep learning network with different loss functions

**Table 1:** The best $F_1$-scores of the deep learning network when trained using each loss function

Loss function	Best $F_1$-score
Dice loss	0.003
Tversky loss ($\alpha =0.6$)	0.663
Tversky loss ($\alpha =0.7$)	0.678
Binary cross-entropy	0.576
Jaccard loss	0.068

## Results and Discussion

### Training Configuration

The deep learning segmentation method was trained on 115 randomly selected patches from the aforementioned annotated patches from both experts and evaluated against the same set of 19 patches that were used to assess expert inter-rater agreement, which were also kept separate from the training data selection pool. The training dataset was drawn entirely from separate tissue cores (and therefore patients) to those used in the validation dataset. Because validation metrics were not used for early stopping, model selection, or anything else aside from extraction of results, the validation set was not kept stratified from the evaluation dataset. We recognize that this would introduce some bias to the hyperparameter tuning process, but only for the high level loss and regularization hyperparameters that were tuned, so the impact and potential for overfitting would be minor. Due to the large size of the patches (480x480 pixels), they were subdivided during training and evaluation. Further details on the patch subdivision are in the appendices (Online Resource 1). For all tests, AdaDelta [17] (a adaptive stochastic gradient descent technique) was used as the optimizer, as this was found to produce good results during network development. The optimizer hyperparameters used were $rho=0.975$ and $learning\_rate=0.005$. A batch size of 4 and input image dimensions of 224x224 pixels were used for all tests.

Since there are readily available ConvNeXt weights which are trained extensively on ImageNet [18], these were used as a starting point for the backbone weights. Given the broad nature of the ImageNet dataset, some of the patterns learned from it were thought likely to be transferable to RNAscope segmentation. The exact weights used were the convnext_base_21k_1k_224_fe weights (pretrained on ImageNet-21k and then on ImageNet-1k, with the final classification layers removed) provided by Sayak Paul on Kaggle [19]. The non-backbone layers were initialized with random weights. Because of the mix of pre-trained weights and newly initialized weights, there was potential for the early epochs of training to destroy the learnt patterns in the pre-trained backbone section. The non-backbone layers would require some epochs to reach useful weights and therefore would not initially allow the backbone layers to be correctly penalized/rewarded based on their ability to recognize useful features. To prevent this loss of feature recognition capability, the backbone layers were locked for the first segment of training, and only the non-backbone layers had their weights trained. Later segments allowed all layers to be trained, which allowed the backbone weights to be fine-tuned. Input images were normalized using the ImageNet normalization statistics to ensure the pre-trained weights were utilized well.

The ground truth masks contained a 5-pixel cross pattern over each RNAscope dot location to mitigate the low positive class representation in the dataset. Stain normalization was not used on the images, since the RNAscope stain in this dataset was so underrepresented that it was removed by stain normalization entirely in many cases. Some data augmentations were also applied during training: rotation of up to 90 degrees and vertical and/or horizontal flips.

**Fig. 12:**
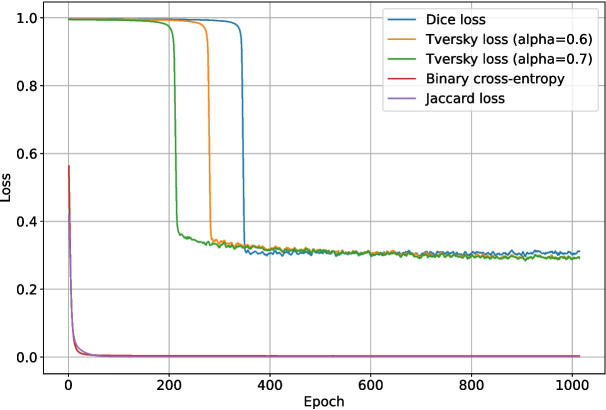
The loss during training of the deep learning network with different loss functions

**Fig. 13:**
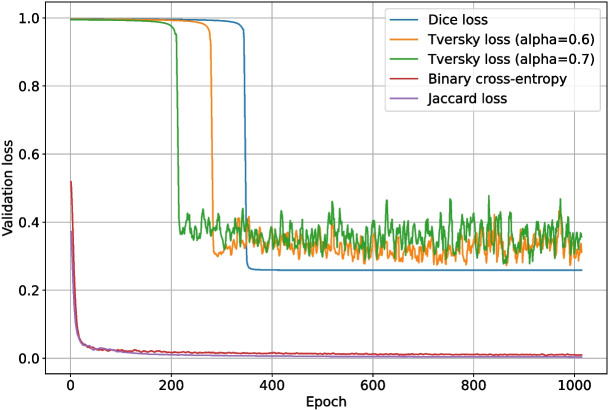
The validation loss during training of the deep learning network with different loss functions

Several sets of tests were run to evaluate which loss function and regularization methods produced the most stable training and best results. The results of these tests are detailed in the following subsections. The results of some less significant sets of tests are instead in the appendices (Online Resource 1).

### Loss Functions

The first set of tests examined loss functions. As we were developing a segmentation network, loss functions that represented the segmentation problem well were sought after. The heavy class imbalance involved in RNAscope segmentation was also taken into consideration; a loss function weighting each pixel and class equally would be unlikely to perform well for this task. Using such a function would likely lead to the network being trained to ignore the positive class entirely and to predict only negatives, as it could obtain a score of more than 99% by that type of metric.

The loss functions selected for comparison were binary cross-entropy, Jaccard loss, Dice loss, and Tversky loss. Binary cross-entropy does not account for class imbalance and was speculated to perform poorly, but was added for the sake of comparison. Jaccard and Dice loss both incentivize overlap (intersection) between predictions and ground truth, while penalizing over-detection. Tversky loss is similar to Dice loss, but allows the user to set the weighting of recall ($\alpha$) and precision ($\beta$) in the formula, which must sum to a total of one. This allows for tuning of sensitivity to match the problem. Dice loss is identical to Tversky loss with $\alpha =0.5$ and $\beta =0.5$. Tversky loss was tested with $\alpha =0.6$ and $\alpha =0.7$ (increased sensitivity/recall).

These tests were run for 1024 epochs, split into two halves. For the first half, the pre-trained ImageNet backbone weights were locked, and only the upscaling layers were trained. For the final half, all layers were trained. DropPath was disabled for the ConvNeXt backbone section, and DropOut ($r=0.15$) was enabled for the upscaling and final layers of the network. The validation intersection over union throughout training for each loss function test is shown in Fig. 11, and the best post-processing $F_1$-score for each test is shown in Table 1. The loss and validation loss during training are shown in Figs. 12 and 13, respectively.

Binary cross-entropy loss performed surprisingly well given that it does not account for class imbalance, weighting each pixel evenly. It was able to reach a validation binary intersection over union score of 0.107 after 1024 epochs, as shown in Fig. 11, and was noticeably more stable than the other loss functions. Intersection over union loss functions are known to be unstable [20], and this is evident in Fig. 11; the Dice loss and Jaccard loss were so unstable that they failed to converge on a good solution. The modified sensitivity weighting of Tversky loss allowed it to converge despite its inherent instability, and its superior representation of the optimization problem at hand allowed it to produce better $F_1$-scores than binary cross-entropy. Although Tversky loss with $\alpha =0.7$ ended with a lower validation intersection over union than binary cross-entropy, its $F_1$-score after post-processing was much higher; it was 0.678, compared to just 0.576 for binary cross-entropy. This implies that Tversky loss learns a more robust representation of the problem, even if its pixel-wise accuracy is not as high. Given the large amount of training instability when using Tversky loss, it was deemed likely that more regularization would improve outcomes. This will be explored in the following sets of tests.

**Fig. 14:**
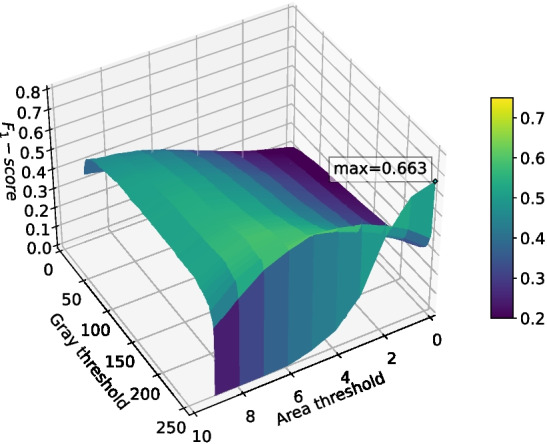
The $F_1$-score of the deep learning network trained with Tversky loss ($\alpha =0.6$) when using differing grey thresholds and area thresholds for segmentation post-processing. The max $F_1$-score is at the area threshold of 0 pixels and grey threshold of 254

**Fig. 15:**
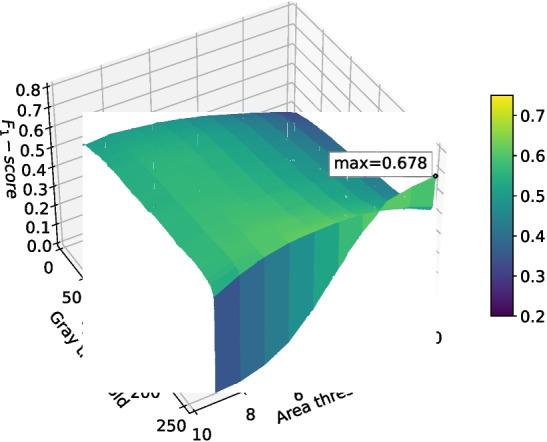
The $F_1$-score of the deep learning network trained with Tversky loss ($\alpha =0.7$) when using differing grey thresholds and area thresholds for segmentation post-processing. The max $F_1$-score is at the area threshold of 0 pixels and grey threshold of 254

The two best performing networks were those that used Tversky loss. Surface plots of the $F_1$-score at different grey threshold and area threshold values (as defined in Section “Post-Processing”) for both networks trained using Tversky loss are shown in Figs. 14 and 15. These decision surface plots demonstrate that these classifiers may not generalize well using the peak threshold values; any deviation in the size of segmentation detections (impacting area threshold) or value (impacting grey threshold) would cause the $F_1$ score to drop significantly. However, both graphs display a larger plateau around area threshold 5, grey threshold 250 which would solve this generalization issue.

While the network using Tversky loss with $\alpha =0.7$ had a slightly higher best $F_1$-score (0.678) than the network using Tversky loss with $\alpha =0.6$ (0.663), its validation intersection over union was less stable and appeared to be over-fitting. Therefore, Tversky loss with $\alpha =0.6$ was selected for use in the remaining tests.

### Backbone Regularization

**Fig. 16:**
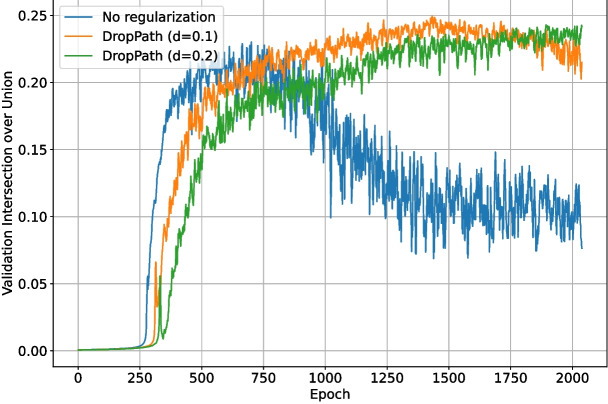
The validation intersection over union during training of the deep learning network with different levels of backbone (downscaling layer) regularization

**Fig. 17:**
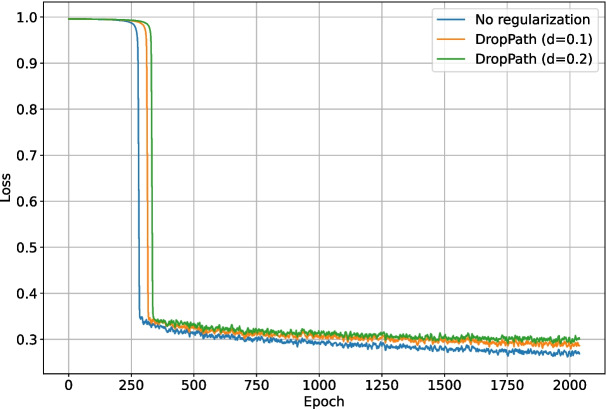
The loss during training of the deep learning network with different levels of backbone (downscaling layer) regularization

**Fig. 18:**
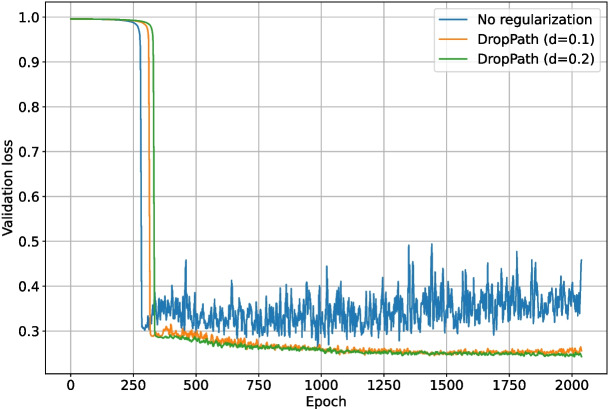
The validation loss during training of the deep learning network with different levels of backbone (downscaling layer) regularization

The second set of tests examined the impact of enabling the DropPath regularization layers existing in the ConvNeXt backbone. As with the previous loss function test, DropOut ($r=0.15$) was enabled for the upscaling and final layers of the network. The backbone layers were locked for the first half of training again, but the total number of epochs was increased to 2048 to ensure any over-fitting would be evident. Backbone DropPath values of 0.0, 0.1, and 0.2 were tested. The validation intersection over union during training is shown in Fig. 16. The best $F_1$-score for each test is shown in Table 2. The loss and validation loss during training are shown in Figs. 17 and 18, respectively.

Enabling DropPath in the backbone layers improved training performance in terms of validation intersection over union, validation loss, and $F_1$-score. Heavier regularization resulted in slower convergence but better performance by the end of training. The performance increase was expected, given the unstable nature of Tversky loss as an intersection-over-union based metric, which was also demonstrated in Fig. 11. Over the increased 2048 epoch training period, the network with DropPath $d=0.0$ was clearly overfitting, and the network with $d=0.1$ was beginning to overfit and decrease in validation intersection over union by the end of training. For this length of training, DropPath $d=0.2$ appeared to work very well, as it was the most stable, did not overfit, and performed the best by the end of training. Increasing DropPath further would result in slower training convergence. DropPath $d=0.2$ for the backbone layers was used for the remaining tests.

**Table 2:** The best $F_1$-scores of the deep learning network when trained using different backbone DropPath probabilities

Backbone regularization	Best $F_1$-score
DropPath ($d=0.1$)	0.681
DropPath ($d=0.2$)	0.712
None	0.649

**Fig. 19:**
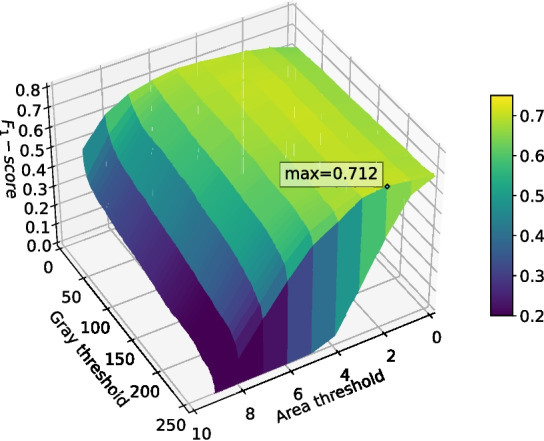
The $F_1$-score of the deep learning network trained with backbone DropPath ($d=0.2$) when using differing grey thresholds and area thresholds for segmentation post-processing. The max $F_1$-score is at the area threshold of 2 pixels and grey threshold of 253

The $F_1$-score for the network trained with backbone DropPath of 0.2 at different area threshold and grey threshold values is shown in Fig. 19. As expected, this shows that this more heavily regularized segmentation network produces more robust and generalizable classifications; minor changes to size of detections (impacting area threshold) or major changes to value (impacting grey threshold) would not significantly change the $F_1$-score.

### Upscaling Node Regularization

The next set of tests examined the viability of using DropPath regularization for the upscaling nodes (the nodes coloured red on Fig. 5) instead of DropOut. The network only converged with DropOut; further details on this set of tests are included in the appendices (Online Resource 1). Additionally, details on an inconclusive set of tests on regularization of the final layers (as depicted in Fig. 4) are also contained in the appendices (Online Resource 1).

### Generated Data

The final set of tests investigated the effect of using artificial training data generated by the method described in Section “Data Generation”. This data generation method was used to generate 1071 patches for use as additional training data. Given the simpler nature of the generated images, they were used to make the early phases of training easier and entirely removed by the end of training. The exact amount of generated samples at each phase of training for this set of tests is shown in Table 3. Each of the 115 real patches was sampled 4 times per epoch to give 460 real samples each epoch, whereas each generated patch was only sampled once, with the patch count varying each phase. The backbone layer weights of the network were only locked for phase 1.

**Fig. 20:**
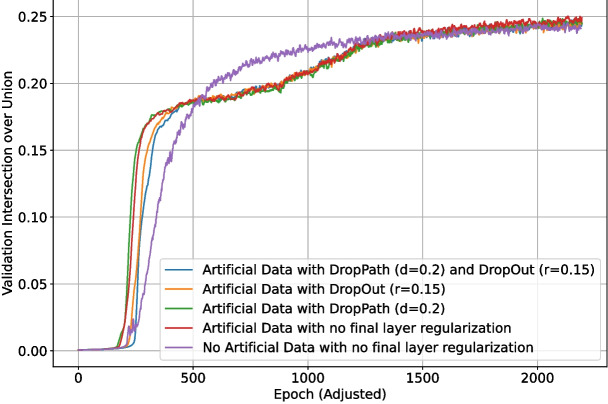
The validation intersection over union during training of the deep learning network using generated data and differing final section regularization methods. Due to the larger number of patches per epoch in early phases of tests using artificial data, the epoch data has been standardized to a length of 460 training samples per epoch for these runs to make them directly comparable with the test with no artificial data

**Fig. 21:**
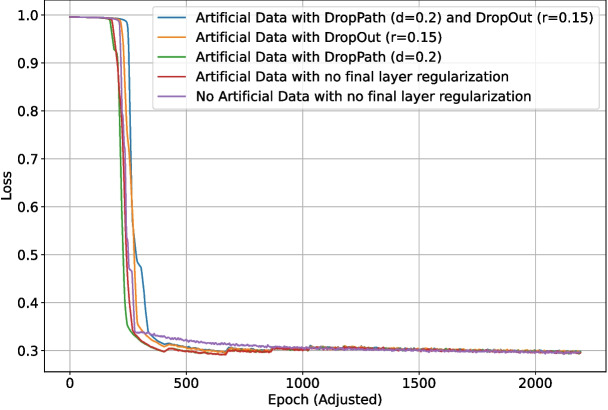
The loss during training of the deep learning network using generated data and differing final section regularization methods. Due to the larger number of patches per epoch in early phases of tests using artificial data, the epoch data has been standardized to a length of 460 training samples per epoch for these runs to make them directly comparable with the test with no artificial data

**Fig. 22:**
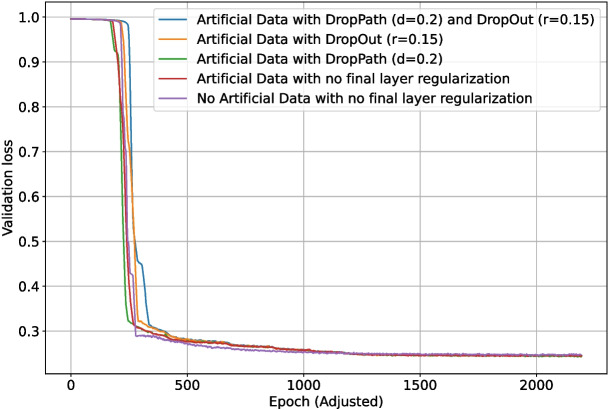
The validation loss during training of the deep learning network using generated data and differing final section regularization methods. Due to the larger number of patches per epoch in early phases of tests using artificial data, the epoch data has been standardized to a length of 460 training samples per epoch for these runs to make them directly comparable with the test with no artificial data

As previously discussed, DropOut ($r=0.15$) was enabled for the upscaling layers of the network. Different regularization methods for the final section of the network (as shown in Fig. 4) were also tested: DropOut $r=0.15$, DropPath $d=0.1$, DropPath $d=0.2$, and no regularization. Due to the inconclusive nature of the previous final section regularization test (in the appendices), each test was run 10 times, and the metrics were then averaged for each set of parameters. One test using no generated data and equivalent training length was added for comparison. The validation intersection over union during training is shown in Fig. 20. The best $F_1$-score for each test is shown in Table 4. The loss and validation loss during training are shown in Figs. 21 and 22, respectively.

**Fig. 23:**
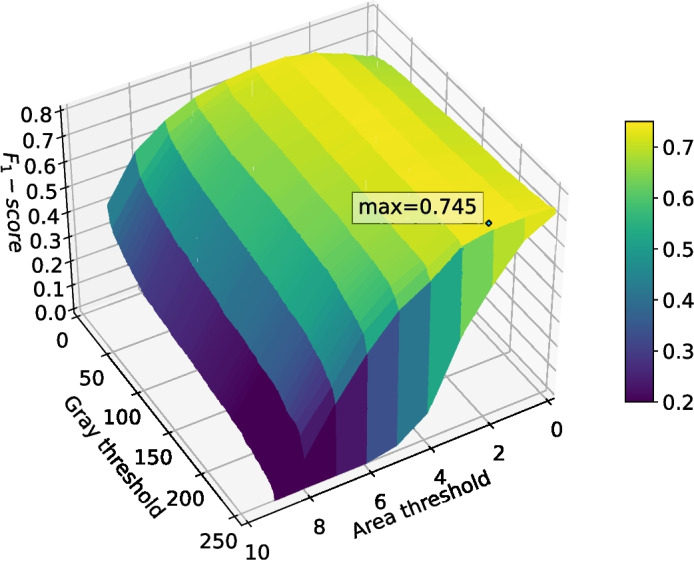
The $F_1$-score of one instance of the deep learning network trained with generated data and no final section regularization when using differing grey thresholds and area thresholds for segmentation post-processing. The max $F_1$-score is at the area threshold of 2 pixels and grey threshold of 246

The $F_1$-score for one instance (out of 10) of the highest performing configuration (using generated data and no final section regularization) at different area threshold and grey threshold values is shown in Fig. 23. Although there was a small amount of variation between instances, the decision surface for this instance shows good generalizability in both area threshold and grey threshold, which correspond to generalizability in detection size and intensity, respectively. The $F_1$-score only begins to significantly drop at area thresholds of 4 or higher and is stable across any grey threshold.

Inclusion of the generated data facilitated much faster convergence initially, with the no artificial data tests consistently performing worse in the first 500 epochs. This changed around 1000 epochs in, with the no artificial data tests having converged better at this time. By the end of training, the artificial data tests again managed to converge better, which is also evident in their higher final $F_1$-score. Including the simpler generated data appears to have been helpful for boosting performance, and altering the training phase scheme to shorten or omit phases 3–5 could help to prevent the slow training around epoch 1000. This would come with the risk of destabilizing the training process since the change in training data would happen less gradually, but having less (albeit more major) changes in training set could also increase stability. The average $F_1$-score for the tests with no final section regularization was the highest, implying that there is sufficient and more effective regularization conducted in the earlier layers of the network.

A final test was conducted to evaluate the performance of the network when trained entirely on one expert’s annotated data and then tested against the other expert’s annotated data. Any patches with annotations by both experts were used in the training dataset, with the corresponding expert’s annotations used as ground truth. This resulted in one test having a training dataset of 113 patches and a test dataset of 31 patches and the other test having a training dataset of 50 patches and a test dataset of 94 patches. This test was intended to assess how well the deep learning network could generalize the subjective annotations of a single expert; however, the results should be interpreted as indicative only. This is because the network’s capability to generalize would be limited when trained using only a single expert’s data, especially with as few as 50 training images. The results of these tests are shown in Table 5. Generated data was used according to the training phases in Table 3.

**Table 3:** The amount of each type of training sample used in each phase of network training when using generated data

Phase	Real samples	Generated samples	Epochs
1	460	1071	128
2	460	460	128
3	460	230	128
4	460	115	128
5	460	58	128
6	460	0	1024

**Table 4:** The best $F_1$-scores of the deep learning network when trained using generated data and differing final section regularization methods

Final section regularization	Best $F_1$-score	Generated data used
DropOut ($r=0.15$)	0.737	Yes
DropPath ($d=0.1$)	0.737	Yes
DropPath ($d=0.2$)	0.740	Yes
None	0.745	Yes
None	0.722	No

The first generalization test, which had a much larger amount of available training data, showed decent performance with an $F_1$-score of 0.624. The second test showed much worse performance, with an $F_1$-score of only 0.314. Overall, these results show that the deep learning network has the potential to learn features that generalize well, but that it cannot train well on as few as 50 training patches.

### Comparison to Literature

Most of the existing methods for RNAscope dot segmentation are not properly automated or do not fully segment RNAscope dots, instead leaving some groups as clusters [10]. The commercial methods do not have a verified accuracy for correct identification of RNAscope dot positions, and the mechanisms by which they operate are not in the public domain. Compared to these existing methods, our deep learning method offers a fully automated approach with no user configuration required, which segments every RNAscope dot rather than leaving some groups as clusters. The feature-based segmentation method by Davidson et al. [13] offered an automated, full RNAscope dot segmentation and therefore was directly comparable to our method. Table 6 shows a comparison of our method to expert performance and to the grey level texture feature method developed by Davidson et al. Our deep learning method outperformed both other methods by a considerable margin.

## Conclusion

This paper covered the development and optimization of a novel deep learning network for accurately segmenting chromogenic RNAscope staining from breast cancer tissue. This network utilized a ConvNeXt backbone, with custom, highly regularized blocks used for subsequent upscaling. Several hyperparameters were optimized to give significantly improved performance. A data generation method was also designed to provide additional training data and was shown to improve final segmentation performance. The final network was able to function well with little training data and was robust to overfitting. It was able to correctly identify RNAscope dots with an $F_1$-score of 0.745, outperforming the expert inter-rater agreement $F_1$-score of 0.596. Our study demonstrates the potential utility of automated RNAscope segmentation and quantification methods which, with further development and validation, would be needed to add RNAscope and other similar technologies into routine pathology workflows.

## Future Work

One aspect of the study that we think could be improved in future work is the assessment metric, which was $F_1$-score based on matching pairs within 5 pixels. We think that a fairer metric would be to count the number of detected RNAscope dots within each cell and use the number of dots in each cell to assign a gene expression classification to that cell (e.g. 0 = none, 1 = low, 2–3 = medium, 4+ = high). Then, the classification agreement across each cell between predictions and ground truth could be assessed to give an accuracy score. This approach would have the advantage of adding some leniency (dots would only need to be within the same cell as their ground truth counterpart), while still assessing the localization of dot detection, which is important since the RNAscope dot density may be different in tumour and non-tumour regions of the tissue image. A limitation of this approach is that it would require nucleus segmentation and subsequent nuclear boundary extrusion to find the boundaries of each cell.

**Table 5:** A comparison of the $F_1$-scores of the deep learning network when trained on the patches annotated by one expert and tested on the patches annotated by the other expert (excluding those patches that were annotated by both experts)

Number of training patches	Number of test patches	$F_1$-score
113	31	0.624
50	94	0.314

Generated data was used to aid the training process, with backbone DropPath rate set to 0.2, upscaling layer DropOut rate set to 0.15, and no DropPath or DropOut regularization in the final layers

**Table 6:** A comparison of the $F_1$-scores of different methods on the same dataset

Method	$F_1$-score
Experts	0.596
Davidson et al. [13]	0.571
This paper	0.745

Another limitation of this study was the limited availability of annotated training data. Only 144 annotated patches of breast cancer tissue from 133 unique patients were available, and all tissue used was from archival FFPE samples. The annotations were done by two experts. A more robust dataset including fresh tissue samples, more expert annotators, or more annotated patches of data would improve the generalizability of the model.

## Data Availability

The data used in this study, which was sourced from tissue samples provided by kConFab, is not permitted to be made publicly available.
